# SNS-Toolbox: An Open Source Tool for Designing Synthetic Nervous Systems and Interfacing Them with Cyber–Physical Systems

**DOI:** 10.3390/biomimetics8020247

**Published:** 2023-06-10

**Authors:** William R. P. Nourse, Clayton Jackson, Nicholas S. Szczecinski, Roger D. Quinn

**Affiliations:** 1 Department of Electrical, Computer, and Systems Engineering, Case Western Reserve University, Cleveland, OH 44106, USA; 2 Department of Mechanical and Aerospace Engineering, Case Western Reserve University, Cleveland, OH 44106, USA; 3 Department of Mechanical and Aerospace Engineering, West Virginia University, Morgantown, WV 26506, USA

**Keywords:** synthetic nervous system, conductance-based modeling, neural simulator, GPU, CUDA, neurorobotics, Python, software, open-source

## Abstract

One developing approach for robotic control is the use of networks of dynamic neurons connected with conductance-based synapses, also known as Synthetic Nervous Systems (SNS). These networks are often developed using cyclic topologies and heterogeneous mixtures of spiking and non-spiking neurons, which is a difficult proposition for existing neural simulation software. Most solutions apply to either one of two extremes, the detailed multi-compartment neural models in small networks, and the large-scale networks of greatly simplified neural models. In this work, we present our open-source Python package SNS-Toolbox, which is capable of simulating hundreds to thousands of spiking and non-spiking neurons in real-time or faster on consumer-grade computer hardware. We describe the neural and synaptic models supported by SNS-Toolbox, and provide performance on multiple software and hardware backends, including GPUs and embedded computing platforms. We also showcase two examples using the software, one for controlling a simulated limb with muscles in the physics simulator Mujoco, and another for a mobile robot using ROS. We hope that the availability of this software will reduce the barrier to entry when designing SNS networks, and will increase the prevalence of SNS networks in the field of robotic control.

## 1. Introduction

A common goal of neuroscientists and roboticists is to understand how animal nervous systems interact with biomechanics and their environment and generate adaptive behavior [1]. By understanding and modeling aspects of the nervous system, it is hoped that robots will be able to exhibit embodied intelligence [2] and exhibit animal-like robustness and adaptability [3]. One approach is to design Synthetic Nervous Systems (SNS), networks of conductance-based neurons and synapses which can be used to model animal nervous systems [4,5] and control robots [6,7,8]. Some strengths of SNS networks include that they can be tuned using analytic design rules [9,10] and that results obtained controlling robotic hardware can propose neurobiological hypotheses [11,12].

In order to design SNS networks for robotic control, software tools are needed. Software for simulating SNS networks should support conductance-based modeling of neurons and synapses, as there are elements of neural behavior in conductance-based models which are incompatible with current-based models [9,13]. Bidirectional synaptic links, such as electrical synapses, should also be supported [14]. Simulators should also support networks with heterogeneous neural models, potentially containing both spiking and non-spiking neurons [7]. While individual spiking neurons can be more computationally powerful than non-spiking neurons [15], non-spiking neurons are capable of capturing much of the dynamics of populations of spiking neurons while being more amenable to real-time simulation [16]. Networks should be able to be constructed in a programmatic way, in order to aid the design of large but formulaic networks [17]. SNS networks should be able to be simulated with faster than real-time performance using CPUs and GPUs, and the same networks should be easily interfaced with physics simulation engines and robotic hardware. Additionally, for accessibility and ease of use in laboratory and educational settings, a simulator software should be cross-platform compatible with the Windows (trademark Microsoft Corporation, Redmond, WA, USA), MacOS (trademark Apple Corporation, Cupertino, CA, USA), and Linux operating systems. A selected survey of available simulation software is presented in Table 1.

Software for simulating conductance-based neural dynamics have long been available, with the most popular options being NEURON [18], NEST [19], GENESIS [20], and Brian [21]. These simulators are capable of simulating highly detailed and biologically accurate neural models, however they were originally designed for the purpose of performing digital experiments and collecting data over a long simulation run. As such, interfacing with external software and systems can be challenging [22] and often requires dedicated software for memory management [23]. Additionally, these simulators are limited to being run on conventional CPUs, although some have begun to be adapted for use with GPUs [24].

Other simulators are capable of designing large networks of neurons using principles from machine learning, such as snnTorch [25], SpykeTorch [26], and BindsNET [27]. These simulators are capable of executing at high speed on both CPUs and GPUs, but they do so at the cost of limiting simulations to reduced models of spiking neurons and current-based synapses. Simulators have also been designed to simulate the Izhikevich neuron [28] and other spiking neurons at scale, including CARLSim [29], NEMO [30], GeNN [31], CNS [32], and NCS6 [33]; however they typically do not support hybrid networks of spiking and non-spiking neurons.

Multiple solutions have been developed which combine a neural dynamics simulator with a physics engine. One approach is to combine an existing neural simulator with an existing physics engine using a middleware memory management software. This has been used to combine Brian [21] and SOFA [34] using CLONES [35], as well as NEST [19] with Gazebo [36] using MUSIC [23]. This approach allows users who are comfortable with a specific neural simulator to interface their networks with physics objects. However, it leads to complicated software dependencies which are difficult to translate to other systems. The first integrated system was AnimatLab [37], which allows networks consisting of either a non-spiking or spiking neural model to control user-definable physics bodies. It also comes with an integrated plotting engine, allowing users to run experiments and analyze data within a single application. Numerous models have successfully been controlled with AnimatLab, both in simulation [4,38,39] and with robotic hardware [6,7], however networks cannot be designed in a programmatic way and are difficult to scale to larger networks [17]. The Neurorobotics Platform (NRP) is a large software suite which integrates multiple neural simulators, including Nengo [40] and NEST [19], with Gazebo [36] in a cloud-based simulation environment. The NRP is a comprehensive toolbox which comes with a variety of advanced visualization tools, and has been used successfully for multiple neurorobotic controllers in simulation [41,42]. However, the NRP comes with much overhead and, as such, is unsuited for real-time control of robotic hardware.

In recent years, high-performance robots have been developed which are controlled using networks of spiking neurons [43,44]. These networks achieve state-of-the-art performance, but rely on specialized neuromorphic hardware, such as Intel’s Loihi processors [45], which are not yet widely available. Lava [46] is a relatively recent and promising software solution for designing spiking networks, but it is primarily designed for use with CPUs and Loihi. Currently, the most widely used software for implementing spiking networks and controlling real hardware is Nengo [40], which has achieved impressive results [44]. However, Nengo is optimized for networks designed using the Neural Engineering Framework [47], and can have reduced performance without the use of neuromorphic hardware. One simulator which can simulate networks with a mixture of spiking and non-spiking neurons in real-time or faster is ANNarchy [16], which does so using a C++ code generation system. However, this code generation system which enables high performance comes at the cost of incompatibility with the Microsoft Windows operating system, which reduces its level of accessibility.

Here, we present SNS-Toolbox, an open-source Python package for the design and simulation of synthetic nervous systems. SNS-Toolbox allows users to design SNS networks with a simple interface and simulate them using established numerical processing libraries on consumer-grade hardware. We focus on simulating a specified set of neural and synaptic dynamics, without dedicated ties to a GUI or a physics simulator. This focus allows the SNS-Toolbox to be easily interfaced with other systems, and for a given network design to be able to be reused without modification in multiple contexts. In previous work [48], we presented an initial version of SNS-Toolbox with reduced functionality. Here we explain in detail the expanded neural and synaptic dynamics supported in the toolbox, and describe the workflow for designing and simulating networks. We provide results which demonstrate comparative performance with other neural simulators, and we showcase the use of SNS-Toolbox in two different applications, motor control of a muscle-actuated biomimetic system in Mujoco [49], and navigation control of a robotic system in simulation using the Robotic Operating System (ROS) [50].

## 2. Materials and Methods

Herein we describe the internal functionality of the SNS-Toolbox, how different neurons and synapses are simulated, designed, and compiled by the user. Section 2.1 defines the neural models which are supported in the toolbox, and Section 2.2 does the same for connection types. Section 3.2.3 describes the design process using SNS-Toolbox, and how a network is compiled and simulated.

All software described is written in Python [51], which was chosen due to its ease of development and wide compatibility. Unless otherwise specified, the units for all quantities are as follows, current (nA), voltage (mV), conductance (μS), capacitance (nF), and time (ms).

### 2.1. Neural Models

SNS-Toolbox is designed to simulate a small selection of neural models, which are variations of a standard leaky integrator. In this section, we present the parameters and dynamics of each neural model which can be simulated using SNS-Toolbox.

#### 2.1.1. Non-Spiking Neuron

The base model for all neurons in SNS-Toolbox is the non-spiking leaky integrator, as has been used in continuous-time recurrent neural networks [52]. This neural model can be used to model non-spiking interneurons, as well as approximate the rate-coding behavior of a population of spiking neurons [9]. The membrane potential *V* behaves according to the differential equation

(1) $$C_{m}\cdot \frac{dV}{dt}=-G_{m}\cdot \left(V-V_{rest}\right)+\sum I_{syn}+I_{bias}+I_{app},$$

where $C_{m}$ is the membrane capacitance, $G_{m}$ the membrane conductance, and $V_{rest}$ is the resting potential of the neuron. $I_{bias}$ is an injected current of constant magnitude, and $I_{app}$ is any external applied current. $I_{syn}$ is the current induced via synapses from presynaptic neurons, the forms of which are defined in Section 2.2.

During simulation, the vector of membrane potentials $\vec{V}$ is updated at each step *t* by representing Equation (1) in a forward-Euler step:

(2) $$\vec{V}\left[t\right]\leftarrow \vec{V}\left[t-\Delta t\right]+{\vec{T}}_{m}⊙\left(-{\vec{G}}_{m}⊙\left(\vec{V}\left[t-\Delta t\right]-{\vec{V}}_{rest}\right)+{\vec{I}}_{b}+{\vec{I}}_{syn}+{\vec{I}}_{app}\right),$$

where ⊙ denotes the element-wise Hadamard product, and Δt represents the simulation timestep. $T_{m}$ is the membrane time factor, which is set as $T_{m}\leftarrow \frac{G_{m}\cdot \Delta t}{C_{m}}$.

#### 2.1.2. Spiking Neuron

Spiking neurons in SNS-Toolbox are represented as expanded leaky integrate-and-fire neurons [53], with the membrane depolarization dynamics described in Equation (1) and an additional dynamical variable for a firing threshold θ [10],

(3) $$\tau_{\theta }\frac{d\theta }{dt}=-\theta +\theta_{0}+m\cdot \left(V-V_{rest}\right)$$

where $\tau_{\theta }$ is a threshold time constant, and $\theta_{0}$ is the initial threshold voltage. *m* is a proportionality constant which describes how changes in *V* affect the behavior of θ, with m=0 causing θ to always equal $\theta_{0}$. When the neuron is subjected to a constant stimulus, m>0 results in a firing rate which decreases over time, and m<0 causes a firing rate which increases over time. Spikes are represented using a spiking variable δ,

(4) $$\delta =\left\{\begin{array}{ll} 1, & V\geq \theta \\ 0, & \mathrm{otherwise}, \end{array}\right.$$

which also triggers the membrane potential to return to rest:

(5) $$\mathrm{if}\;\delta =1,V\leftarrow \leftarrow V_{rest}.$$

The vector of firing thresholds $\vec{\theta }$ is updated as

(6) $$\vec{\theta }\left[t\right]\leftarrow \vec{\theta }\left[t-\Delta t\right]+{\vec{T}}_{\theta }\left[t\right]⊙\left(-\vec{\theta }\left[t-\Delta t\right]+{\vec{\theta }}_{0}+\vec{m}⊙\left(\vec{V}\left[t-\Delta t\right]-{\vec{V}}_{rest}\right)\right),$$

where $T_{\theta }=\frac{\Delta t}{\tau_{\theta }}$ is the threshold time factor. Based on the threshold states, the spiking states are updated as

(7) $$\vec{\delta }\left[t\right]\leftarrow sign\left(min\left(0,\vec{\theta }-\vec{V}\right)\right).$$

Note that for simplified implementation, all spikes with SNS-Toolbox are internally represented as impulses of magnitude −1. Using these spike states, the membrane potential of each neuron which spiked is reset to $V_{\mathrm{rest}}$

(8) $$\vec{V}\left[t\right]\leftarrow \left(\vec{V}\left[t\right]-{\vec{V}}_{rest}\right)⊙\left(\vec{\delta }\left[t\right]+1\right)+{\vec{V}}_{rest}.$$

#### 2.1.3. Neuron with Voltage-Gated Ion Channels

The other neural model available within SNS-Toolbox is a non-spiking neuron with additional Hodgkin–Huxley [54] style voltage-gated ion channels. The membrane dynamics are similar to Equation (1), with the addition of an ionic current $I_{ion}$ [55]:

(9) $$C_{m}\cdot \frac{dV}{dt}=-G_{m}\cdot \left(V-V_{rest}\right)+\sum I_{syn}+I_{bias}+I_{app}+I_{ion}.$$

This ionic current is the sum of multiple voltage-gated ion channels, all obeying the following structure:

(10) $$I_{ion}=\sum_{j}G_{ion,j}\cdot a_{\infty ,j}^{p_{a,j}}\left(V\right)\cdot b_{j}^{p_{b,j}}\cdot c_{j}^{p_{c,j}}\cdot \left(E_{ion,j}-V\right).$$

Any neuron within a network can have any number of ion channels. $G_{ion,j}$ is the maximum ionic conductance of the *j^th^* ion channel, and $E_{ion,j}$ is the ionic reversal potential. *b* and *c* are dynamical gating variables, and have the following dynamics

(11) $$\frac{dz_{j}}{dt}=\frac{z_{\infty ,j}\left(V\right)-z_{j}}{\tau_{z,j}\left(V\right)},$$

where functions of the form $z_{\infty ,j}$ are a voltage-dependent steady-state

(12) $$z_{\infty ,j}\left(V\right)=\frac{1}{1+K_{z,j}\cdot \exp\left(S_{z,j}\cdot \left(E_{z,j}-V\right)\right)},$$

and $\tau_{z,j}$ is a voltage-dependent time constant

(13) $$\tau_{z,j}\left(V\right)=\tau_{max,z,j}\cdot z_{\infty ,j}\left(V\right)\cdot \sqrt{K_{z,j}\cdot \exp\left(S_{z,j}\cdot \left(E_{z,j}-V\right)\right)}.$$

*p* denotes an exponent, and $E_{z,j}$ is the gate reversal potential. $K_{z,j}$ and $S_{z,j}$ are parameters which shape the $z_{\infty ,j}$ and $\tau_{z,j}$ functions. $\tau_{max,z,j}$ is the maximum value of $\tau_{z,j}$. Note that depending on the desired ion channel, the exponent for various sections can be set to 0 in order to effectively remove it from Equation (10). One particular example of this is a neuron with a persistent sodium current, which is also available as a preset in SNS-Toolbox,

(14) $$I_{ion}=\sum_{j}G_{Na,j}\cdot m_{\infty ,j}\left(V\right)\cdot h_{j}\cdot \left(E_{Na,j}-V\right),$$

which is the same as Equation (10) with one dynamic variable disabled and some variable renaming.

### 2.2. Connection Models

Within SNS-Toolbox, neurons are connected using connection objects. These can either define links between individual neurons, or structures of connectivity between neural populations (see Section 2.2.4).

#### 2.2.1. Non-Spiking Chemical Synapse

When connecting non-spiking neurons, non-spiking chemical synapses are typically used. The amount of synaptic current $I_{syn}^{ji}$ to post-synaptic neuron *i* from presynaptic neuron *j* is

(15) $$I_{syn}^{ji}=G_{syn}^{ji}\left(V_{j}\right)\cdot \left(E_{syn}^{ji}-V_{i}\right),$$

where $E_{syn}^{ji}$ is the synaptic reversal potential. $G_{syn}^{ji}\left(V_{j}\right)$ is the instantaneous synaptic conductance, which is a function of the presynaptic voltage $V_{j}$:

(16) $$G_{syn}^{ji}\left(V_{j}\right)=max\left(0,min\left(G_{max,non}^{ji}\cdot \frac{V_{j}-E_{lo}}{E_{hi}-E_{lo}},G_{max,non}^{ji}\right)\right).$$

$G_{max,non}^{ji}$ is the maximum synaptic conductance, and voltages $E_{lo}$ and $E_{hi}$ define the range of presynaptic voltages where the synaptic conductance depends linearly on the presynaptic neuron’s voltage.

When simulating, Equation (15) is expanded to use matrices of synaptic parameters (denoted in bold),

(17) $${\vec{I}}_{syn}\left[t\right]\leftarrow \sum_{j}{G_{\mathrm{syn}}}^{i,j}\left[t\right]\cdot E^{i,j}-\vec{V}\left[t-\Delta t\right]⊙\sum_{j}{G_{\mathrm{syn}}}^{i,j},$$

and each term is summed column-wise to generate the presynaptic current for each neuron. Synaptic parameter matrices have an NxN structure, with the columns corresponding to the presynaptic neurons and the rows corresponding to the postsynaptic neurons. Equation (16) is also expanded to use parameter matrices,

(18) $$G_{\mathrm{non}}\left[t\right]\leftarrow max\left(0,min\left(G_{\max,\mathrm{non}}\cdot \frac{\vec{U}\left[t-\Delta t\right]-E_{\mathrm{lo}}}{E_{\mathrm{hi}}-E_{\mathrm{lo}}},G_{\max,\mathrm{non}}\right)\right).$$

#### 2.2.2. Spiking Chemical Synapse

Spiking chemical synapses produce a similar synaptic current as non-spiking chemical synapses (Equation (15)), but a key difference is that $G_{syn}^{ji}$ is a dynamical variable defined as

(19) $$\tau_{syn}^{ji}\frac{dG_{syn}^{ji}}{dt}=-G_{syn}^{ji}.$$

(20) $$\mathrm{if}\;\delta =1,G_{max,spike}^{ji}\leftarrow G_{syn}^{ji}.$$

The conductance is reset to $G_{max,spike}^{ji}$, the maximum value, whenever the presynaptic neuron spikes. Otherwise, it decays to zero with a time constant of $\tau_{syn}^{ji}$. When simulated, these dynamics are represented as

(21) $$G_{\mathrm{spike}}\left[t\right]\leftarrow G_{\mathrm{spike}}\left[t-1\right]\cdot \left(1-T_{\mathrm{syn}}\right),$$

where $G_{\mathrm{spike}}$ is the matrix of spiking synaptic conductances, and $T_{\mathrm{syn}}$ is the synaptic time factor matrix.

An additional feature available with spiking synapses is a synaptic propagation delay. This sets the number of simulation steps it takes for a spike to travel from one neuron to another using a specific synapse, a feature which is useful for performing some aspects of temporal computation [17]. If the synapse between neurons *j* and *i* has a delay of *d* timesteps, the delayed spike is represented as

(22) $$\delta_{delay}^{ji}\left[t\right]=\delta^{ji}\left[t-d\cdot \Delta t\right].$$

For simulation, this propagation delay is implemented using a buffer matrix $\delta_{\mathrm{buffer}}$ with *N* columns and *D* rows, where *D* is the longest delay within the network. The rows of $\delta_{\mathrm{buffer}}$ are shifted down at each timestep, and the first row is replaced with current spike state vector $\vec{\delta }\left[t\right]$. $\delta_{\mathrm{buffer}}$ is then transformed into a matrix of delayed spikes $\delta_{\mathrm{delay}}$ by rearranging based on the delay of each synapse in the network. $\delta_{\mathrm{delay}}$ is then used to simulate the synaptic reset dynamics from Equation (20),

(23) $$G_{\mathrm{spike}}\left[t\right]\leftarrow max\left(G_{\mathrm{spike}}\left[t\right],-\delta_{\mathrm{delay}}\left[t\right]⊙G_{\max,\mathrm{spike}}\right).$$

#### 2.2.3. Electrical Synapses

Electrical synapses, or gap junctions, are resistive connections between neurons that do not use synaptic neurotransmitters. As a result, the neurons exchange current proportional to the difference between their voltage values. Their current is defined as

(24) $$I_{syn}^{ji}=G_{syn,electrical}\cdot \left(V_{j}-V_{i}\right),$$

where $G_{syn,electrical}$ is the synaptic conductance. Electrical synapses are simulated in SNS-Toolbox using a similar formulation as Equation (17),

(25) $${\vec{I}}_{syn}\left[t\right]\leftarrow \sum_{j}{G_{\mathrm{elec}}}^{i,j}\cdot {\vec{V}}_{last}^{j}-\vec{V}\left[t-\Delta t\right]⊙\sum_{j}{G_{\mathrm{elec}}}^{i,j}.$$

SNS-Toolbox simulates electrical synapses as bidirectional by default, where current can flow in either direction between the connected neurons. Rectified connections are also supported, where current only flows from the presynaptic to the postsynaptic neuron and only if $V_{pre}>V_{post}$. When simulating with rectified electrical synapses, a binary mask M is generated,

(26) $$M\leftarrow H\left({\left({\vec{V}}_{last}⊖{\vec{V}}_{last}\right)}^{T}\right),$$

where ⊖ denotes outer subtraction. The voltage of each neuron is subtracted in a pairwise fashion, with the result processed by the heaviside step function *H*. This generates a matrix where each element is 1 if current is allowed to flow in that direction, and 0 otherwise. This binary mask is then applied to a synaptic conductivity matrix $G_{\mathrm{rec}}$ to obtain the masked conductance $M_{G}$,

(27) $$M_{G}\leftarrow M⊙G_{\mathrm{rec}}.$$

To generate the opposite current flow in rectified synapses, the masked conductance is then added to its transpose with the diagonal entries removed,

(28) $$M_{D}\leftarrow M_{G}+M_{G}^{T}-\mathrm{diag}\left(M_{G}\right).$$

This final masked, transformed conductance matrix $M_{D}$ is substituted for $G_{\mathrm{elec}}$ in Equation (25)

(29) $${\vec{I}}_{syn}\left[t\right]\leftarrow \sum_{j}{M_{D}}^{i,j}\cdot {\vec{V}}_{last}^{j}-\vec{V}\left[t-\Delta t\right]⊙\sum_{j}{M_{D}}^{i,j}.$$

#### 2.2.4. Matrix and Pattern Connections

In their base form, each of the preceding connection models defines the connection between two individual neurons. However, the connections’ behavior can be extended to defining connections between populations of neurons. Following the model presented in [10], in the simplest form of population-to-population connection all neurons become fully connected and the synaptic conductance is automatically scaled such that the total conductance into each postsynaptic neuron is the same as the original synapse.

For more complex desired behavior, more types of population-to-population connections are available. Matrix connections allow the user to specify the exact matrices for each synaptic parameter, and one-to-one connections result in each presynaptic neuron to be connected to exactly one postsynaptic neuron, with all synapses sharing the same properties. Pattern connections are also available, modeled after convolutional connections in ANNs [56]. In pattern connections, a kernel matrix K can be given,

(30) $$K=\left[\begin{array}{ccc} a & b & c\\ d & e & f\\ g & h & i \end{array}\right],$$

where the indices are values for a single synaptic parameter ($G_{max},E_{syn},\mathrm{etc}.$). If K describes the connection pattern between two 3×3 neural populations, then the resulting synaptic parameter matrix P will present the following structure:

(31) $$P=\left[\begin{array}{ccccccccc} e & f & 0 & h & i & 0 & 0 & 0 & 0\\ d & e & f & g & h & i & 0 & 0 & 0\\ 0 & d & e & 0 & g & h & 0 & 0 & 0\\ b & c & 0 & e & f & 0 & h & i & 0\\ a & b & c & d & e & f & g & h & i\\ 0 & a & b & 0 & d & e & 0 & g & h\\ 0 & 0 & 0 & b & c & 0 & e & f & 0\\ 0 & 0 & 0 & a & b & c & d & e & f\\ 0 & 0 & 0 & 0 & a & b & 0 & d & e \end{array}\right]$$

### 2.3. Inputs and Outputs

In order for an SNS to interact with external systems, it must be capable of receiving inputs and sending outputs. For applying external stimulus to a network, input sources can be added. These sources can be either individual elements or a one-dimensional vector, and are applied to the network via ${\vec{I}}_{app}$,

(32) $${\vec{I}}_{app}\left[t\right]\leftarrow C_{\mathrm{in}}\cdot {\vec{I}}_{ext}\left[t\right],$$

where $C_{\mathrm{in}}$ is an LxN binary masking matrix which routes each input to the correct target neuron. L is the number of input elements, and N is the number of neurons in the network. This external input vector is varied from step to step and could come from any source (e.g., static data, real-time sensors).

Output monitors can also be added, both for sending signals to other systems and for observing neural states during simulation. These outputs are assigned one-to-one to each desired neuron, meaning one output applied to a population of five neurons results in five individual outputs. Output monitors can be voltage-based or spike-based, where the output is the direct voltage or spiking state of the source neuron. During simulation, the output vector is computed as

(33) $$\vec{Out}\left[t\right]\leftarrow C_{\mathrm{out},\mathrm{voltage}}\cdot \vec{V}\left[t\right]+C_{\mathrm{out},\mathrm{spike}}\cdot \vec{\delta }\left[t\right],$$

where $C_{\mathrm{out},\mathrm{voltage}}$ and $C_{\mathrm{out},\mathrm{spike}}$ are connectivity matrices for the voltage and spike-based monitors, respectively.

### 2.4. Software Design and Workflow

Using SNS-Toolbox, the design and implementation of an SNS is split across three phases, a design phase, a compilation phase, and a simulation phase.

#### 2.4.1. Design

To design a network, users first define neuron and connection types. These describe the parameter values of the various neural and synaptic models in the network, which can be subsequently reused. Once the neuron and connection presets are defined, they can be incorporated into a Network object (for a complete inventory of the different elements which can be added to a Network, refer to Section 2.1, Section 2.2 and Section 2.3). First, the user can add populations of neurons by giving the neuron type, the size or shape of the population, and a name to refer to the population. When simulated, all populations will be flattened into a one-dimensional vector, but during the design process they can be represented as a two-dimensional matrix for ease of interpretation (e.g., working with two-dimensional image data). After populations are defined and labeled, the user can add synapses or patterns of synapses between neurons/populations, giving an index or character-string corresponding to the source and destination neurons or populations and the connection preset.

Once a network is designed, it can also be used as an element within another network. In this way, a large network can be designed using large collections of predefined subnetworks, in a methodology referred to as the Functional Subnetwork Approach (FSA). Available within the SNS-Toolbox is a collection of subnetworks which perform simple arithmetic and dynamic functions. For a complete explanation of these networks, as well as the FSA, please refer to [9].

#### 2.4.2. Compilation

While it describes the full structure of an SNS, a Network object is merely a dictionary which contains all of the network parameters. In order to be simulated, it must be compiled into an executable state. Given a Network, the SNS-Toolbox can compile a model capable of being simulated using one of the four software backends, NumPy [57], PyTorch [58], a PyTorch-based sparse matrix library (torch.sparse), and an iterative evaluator which evaluates each synapse individually. These backends are all built on well-established numerical processing libraries, with PyTorch bringing native and simple GPU support. Each backend has different strengths and weaknesses, which are illustrated in Section 3.2.2. Although each is different, all backends are compiled following the general procedure described in Algorithm 1. Once a network is compiled, it can either be immediately used for simulation or saved to disk for later use.
**Algorithm 1** General compilation procedure.**function** Compile(net, Δt)    Get network parameters    Initialize state and parameter vectors and matrices    Set parameter values of each neuron in each population    Set input mapping and connectivity parameter values    Set connection synaptic parameter values    Calculate time factors    Initialize propagation delay buffer    Set output mapping and connectivity parameter values    **return** model**end function**

#### 2.4.3. Simulation

**Figure 1:**
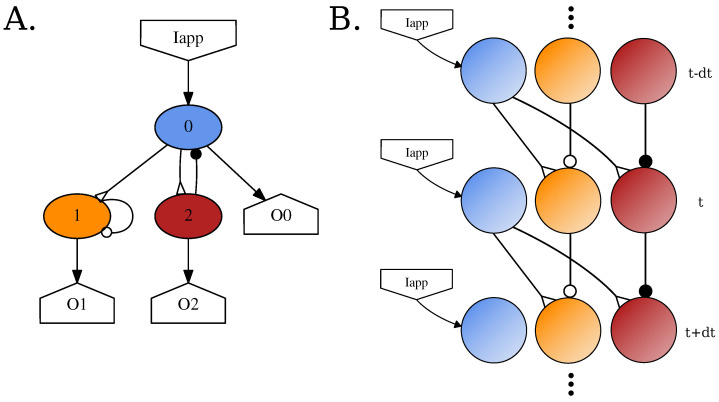
Simulation method for a small example network using the SNS-Toolbox. (**A**) Overall network diagram generated within the toolbox. (**B**) Diagram of the general computational flow when simulating the network. The network is unfolded in time, and neural voltages are propagated in feedforward layers from one time-step to another.

Since the SNS-Toolbox focuses on smaller networks which are connected with varying levels of feedback loops [6,7,52,59,60] instead of multiple massively connected layers [61], we optimize our computations by representing all networks as single-layer, fully-connected recurrent networks. During simulation, the neural dynamics are evaluated by unfolding the network through time. This is similar to the method developed by Werbos et al., for training recurrent ANNs [62]. See Figure 1 for a visual representation of this strategy. At each timestep, every neuron can receive input from any neuron at the previous step (including itself via an autapse [63]). Although the SNS-Toolbox only acts as a neural dynamics simulator, it is extensible to interact with other systems for controlling robot (Section 3.3) or musculoskeletal (Section 3.4) dynamics.

## 3. Results

In this Section, we provide results showcasing the capabilities of SNS-Toolbox. We first provide quantitative benchmarks which characterize the performance of the software, and conclude with two application examples.

### 3.1. Specifications

Unless otherwise specified, all of the following results were obtained using the software and hardware presented in Table 2.

### 3.2. Performance Benchmarking

For evaluating the performance of the SNS-Toolbox, we present benchmarking results for varying network size, structure, and type. In these benchmarks, networks consist entirely of either spiking or non-spiking neurons, and are either densely or sparsely connected. In densely connected networks, every neuron is synaptically connected to every other neuron. For sparse networks, the neurons are connected with the following structure; 8% of neurons receive external input, 12% of neurons are recorded for output, and the number of neurons and synapses is equal. This structure is based on general principles observed in previous large-scale synthetic nervous systems [6,7].

#### 3.2.1. Maximum Network Size

Networks were constructed following the structure described in Section 3.2, and increased in size until one of the following two termination conditions were met, either the network parameter matrices could not fit in memory or network synthesis took an excessive amount of time (≥10 h). These experimental results are shown in Table 3. The limiting factors of whether a network can successfully be synthesized are the synaptic parameter matrices, as these increase in size quadratically as the size of the network increases. CPU-based backends are able to achieve the highest network sizes, which is expected due to the increased volume of memory available to the CPU. The iterative backend is able to achieve the highest sizes of network, since its neural and synaptic dynamics are computed by iterating over one-dimensional arrays instead of vector and matrix operations on two-dimensional arrays. All of the sparse networks took significantly longer to synthesize, resulting in termination of their testing before running out of memory.

#### 3.2.2. Backend Performance

**Figure 2:**
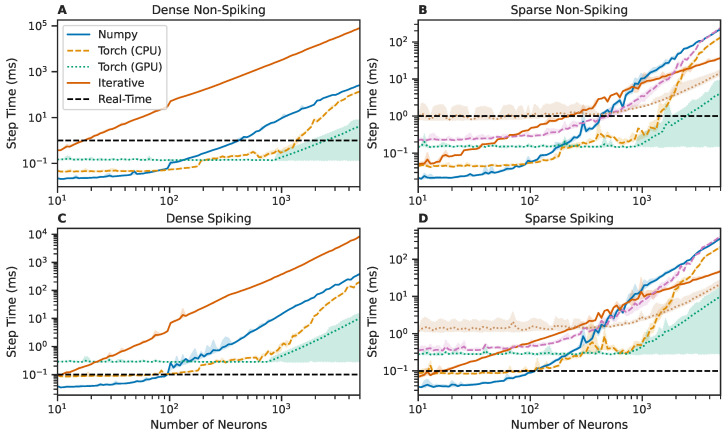
Comparison of wall-clock times to simulate a network for one simulation time-step over varying network sizes, using the six software backends provided in SNS-Toolbox. (**A**,**B**): Networks of non-spiking neurons, (**C**,**D**): networks of spiking neurons. **Left**: Fully-connected networks, **Right**: Sparsely connected networks, following the structure described in Section 3.2. Lines denote the mean over 1000 steps, shaded region denotes the area between the fifth and ninety-fifth percentiles. The real-time limit is denoted with a horizontal dashed black line.

We show that SNS-Toolbox is capable of simulating thousands of non-spiking neurons in real-time or faster, with slower performance when simulating spiking neurons. In total, 100 networks, which varied in size from 10 to 5000 neurons in a logarithmic spacing, were generated and simulated for 1000 steps in each backend. A simulation step of Δt=0.1ms was used. The elapsed time to simulate each step was recorded, and the results are shown in Figure 2. Each of the available backends exhibit different strengths and weaknesses. For networks with less than 100 neurons, the Numpy [57] backend runs the fastest, followed by the PyTorch [58] backend running on the CPU. Once networks increase in size beyond 200–300 neurons, the PyTorch backend running on a GPU becomes the fastest. While this backend is the fastest, the exchange of data between the CPU and GPU results in a higher degree of temporal variability than the CPU-based backends. Further investigation is needed to reduce this variability in performance.

The exact threshold for what could be considered real-time performance is dependent on the simulation step size, which, in turn, is dependent on the membrane properties of neurons within the network. While all networks in this test were simulated with the same step size for consistency, accurately simulating spiking networks will generally require a finer simulation step than non-spiking networks. In this test, an elapsed time of 0.1 ms per step is considered real-time for the spiking networks. A step size of 1 ms would suffice for the non-spiking networks tested in this section, so their real-time limit is 1 ms. For non-spiking networks, this means that networks up to about 3000 neurons can be simulated in real-time, and for spiking networks the threshold is about 100–200 neurons.

#### 3.2.3. Benchmarking Alternative Software

The SNS-Toolbox is faster than the majority of similar neural simulators. We perform the same testing procedure presented in Section 3.2.2, and compare against the behavior of similar simulators, namely Brian2 [21], Nengo [40], and ANNarchy [16]. For these other simulators, the neural and synaptic dynamics for basic spiking and non-spiking neurons within SNS-Toolbox (see Section 2.1 and Section 2.2) were implemented and verified to match the behavior in SNS-Toolbox. In Brian2 and ANNarchy, this was completed via their built-in interfaces for interpreting custom behavioral strings. This process was less straightforward in Nengo, requiring a custom Nengo process object which re-implemented the equations as performed in SNS-Toolbox. As such, while the networks are able to successfully run in Nengo, they are not fully compatible with the rest of the Nengo ecosystem. Since these benchmarks are not being compared against biological recordings, validation is completed by comparing the behavior of the neural models across simulators and verifying that the simulation recordings are identical.

**Figure 3:**
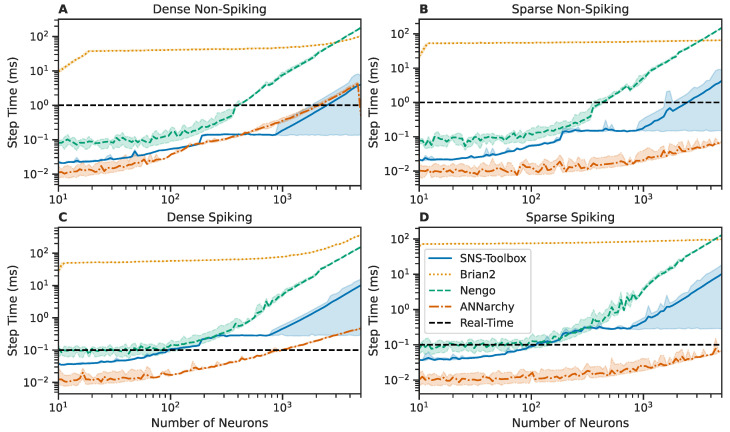
Comparison of wall-clock times for SNS-Toolbox to simulate a network for one simulation time-step over varying network sizes, using SNS-Toolbox and three other neural simulators (Brian2 [21], Nengo [40], and ANNarchy [16]). For the following simulators, the time data presented are chosen as the best-performing backend variant, Brian2, standard Brian2, and the GPU-accelerated Brian2CUDA; SNS-Toolbox, all available variants; and ANNarchy, CPU-based compilation, and GPU-based compilation. (**A**,**B**): Networks of non-spiking neurons, (**C**,**D**): networks of spiking neurons. **Left**: Fully-connected networks, **Right**: Sparsely connected networks, following the structure described in Section 3.2. Lines denote the mean over 1000 steps, shaded region denotes the area between the fifth and ninety-fifth percentiles. The real-time limit is denoted with a horizontal dashed black line.

Results are shown in Figure 3. For clarity, simulators with multiple backends or variants are condensed to show the best performing version for each network size. The variants tested in Brian2 are the normal version on CPU, and the GPU-accelerated Brian2CUDA [24], and ANNarchy was compiled using the CPU and GPU paradigms. All SNS-Toolbox backends were tested. Across all network sizes and structures, SNS-Toolbox is faster or within performance variance of Brian2 and Nengo. SNS-Toolbox is faster than ANNarchy for some densely-connected non-spiking networks, but, in general, is slower but competitive across the test suite. Suggestions for improving this speed discrepancy will be explored in the Discussion.

#### 3.2.4. Performance on Embedded Hardware

**Figure 4:**
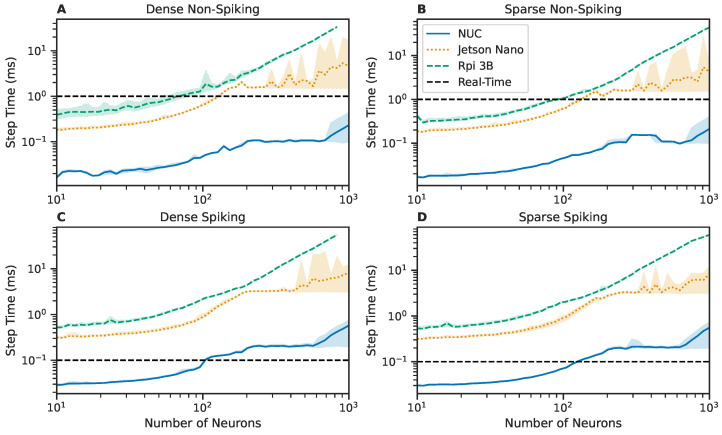
Comparison of wall-clock times to simulate a network for one simulation time-step over varying network sizes, using SNS-Toolbox on three different embedded computing platforms (Intel NUC, Raspberry Pi version 3b, and an NVIDIA Jetson Nano). The time data presented are chosen as the best-performing backend variant at each network size, with GPU-based backends excluded on the Raspberry Pi. (**A**,**B**): Networks of non-spiking neurons, (**C**,**D**): networks of spiking neurons. **Left**: Fully-connected networks, **Right**: Sparsely connected networks, following the structure described in Section 3.2. Lines denote the mean over 1000 steps, shaded region denotes the area between the fifth and ninety-fifth percentiles. The real-time limit is denoted with a horizontal dashed black line.

The testing procedure presented in Section 3.2.2 is again repeated, testing the performance of SNS-Toolbox on various embedded computing platforms. These included a Raspberry Pi Model 3B (trademark Raspberry Pi Limited, Cambridge, UK), Jetson Nano 4GB (trademark NVIDIA Corporation, Santa Clara, CA, USA), and an Intel NUC SWNUC11PHKi7c00 (trademark Intel Corporation, Santa Clara, CA, USA) with 32 GB of RAM. Due to the reduced available memory available on the Raspberry Pi and Jetson, network size is varied logarithmically from 10 to 1000 neurons, instead of the 10–5000 neurons in Section 3.2.2 and Section 3.2.3. Results are shown in Figure 4; for clarity, all backends are condensed for each device such that the best performing solution at each network size is presented. The Raspberry Pi performs comparably with a Jetson Nano, with the Jetson exhibiting slightly better performance across all network sizes. The amount of memory available on the Raspberry Pi is the smallest of the three devices, so it is unable to simulate densely-connected networks over approximately 900 neurons in size. The Intel NUC is a significantly more powerful computing platform than the Raspberry Pi or the Jetson Nano, and accordingly behaves more closely to desktop-level performance.

### 3.3. Mobile Robot Control

**Figure 5:**
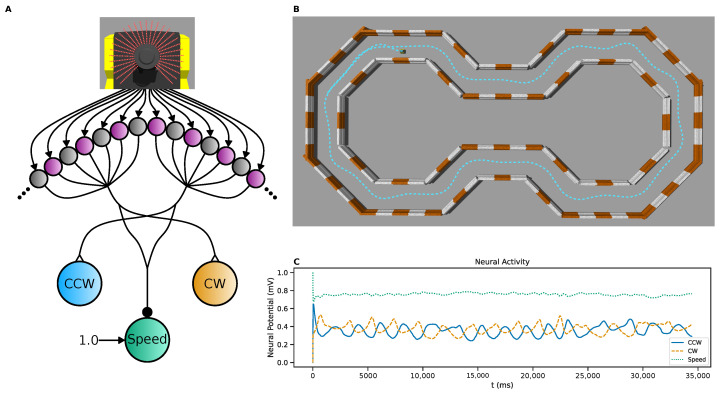
LiDAR-based steering algorithm for a simulated mobile robot using ROS. (**A**): Network diagram of the control network. Each distance measurement angle of a simulated LiDAR is inverted, scaled, and mapped as the input to a single input processing neuron. These are then summed onto directional neurons corresponding to clockwise or counter-clockwise rotation, depending on which half of the scanning field the neuron represents. All sensory neurons also connect to a speed control neuron. The difference between the directional neurons is taken as the rotational velocity, and the speed control neuron is scaled by the maximum speed to control the linear velocity. (**B**): Overhead view of the simulation environment in Gazebo [36]. Orange and white barriers act as boundaries of the course, the robot trajectory is superimposed on top with a dashed blue line. (**C**): Neural activity of the three command neurons during the generation of the trajectory shown above.

As a toy application example, we use SNS-Toolbox to control a simulated mobile robot. A skid-steer Jackal robot (trademark Clearpath Robotics, Kitchener, ON, USA) is placed in a navigational course resembling a figure-eight in the Gazebo physics simulator [36] (Figure 5B), with the goal being to drive the robot around the course without colliding with any of the walls or barriers. The simulated robot is equipped with a planar LiDAR unit, and is controlled and operated using the ROS software ecosystem [50]. We implement the neural control system as a ROS node which subscribes to the angular distance readings from the laser scanner, and publishes to the velocity controller onboard the robot.

The control network, shown in Figure 5A, implements a Braitenberg-inspired [64] steering algorithm. The laser scan sends distance measurements for 720 points in a 270${}^{\circ }$ arc around the front of the robot, and each neuron in a population of 720 non-spiking neurons receives external current from a corresponding directional distance scan. These currents are scaled and mapped by the following relationship,

(34) $$I_{app}\left(D\right)=\frac{D^{-1}-D_{max}^{-1}}{D_{min}^{-1}-D_{max}^{-1}},$$

such that each neuron has a steady-state voltage of 0 when the sensor distance *D* is at its maximum value $D_{max}$, and increases to 1 when the distance is at its minimum $D_{min}$. This population then excites two heading control neurons, with the left 360 neurons exciting the clockwise rotation neuron, and the right 360 exciting the counter-clockwise rotation neuron. All synapses between the sensory and heading control neurons share the same synaptic conductance. The difference between the potentials of these two neurons is taken and scaled to generate the desired angular velocity of the robot,

(35) $$\nu_{ang}=K_{ang}\cdot \left(V_{CW}-V_{CCW}\right).$$

As the robot approaches a barrier, the system generates stronger rotational commands to move away from the obstacle. All 720 sensory neurons also inhibit a speed control neuron, which scales the linear velocity of the robot as

(36) $$\nu_{lin}=\nu_{lin,max}\cdot V_{Speed}.$$

The speed control neuron also has a constant applied bias current of 1 nA. This has the effect of dynamically slowing the robot as it becomes closer to obstacles, allowing the rotational commands to correctly orient the robot. This controller results in successful navigation of the driving course in 133.24 s, with minimal tuning. Neural parameter values can be found in Table A1, synaptic parameter values in Table A2, and mapping and simulation parameter values in Table A3. Currently the velocity is updated with every neural step, however for improved speed performance the velocity can be updated after multiple neural steps. This allows the neural states to converge to a steady-state for each scan distance, and reduces the amount of communication traffic.

Braitenberg-inspired [64] networks have been widely used for steering and lane-keeping tasks in the past [65,66,67] to great success. The network designed in this section is intended as a proof of concept to showcase the ability to interface SNS-Toolbox with ROS simulations, not as a state-of-the-art steering algorithm.

### 3.4. Musculoskeletal Dynamics

In Deng et al. [68], an SNS was designed to control a biomechanical simulation of rat hindlimbs, with the network and body dynamics simulated using AnimatLab [37]. Here we reimplement this SNS using SNS-Toolbox and interface it with a new biomechanical model implemented in the physics simulator Mujoco [49].

#### 3.4.1. Neural Model

**Figure 6:**
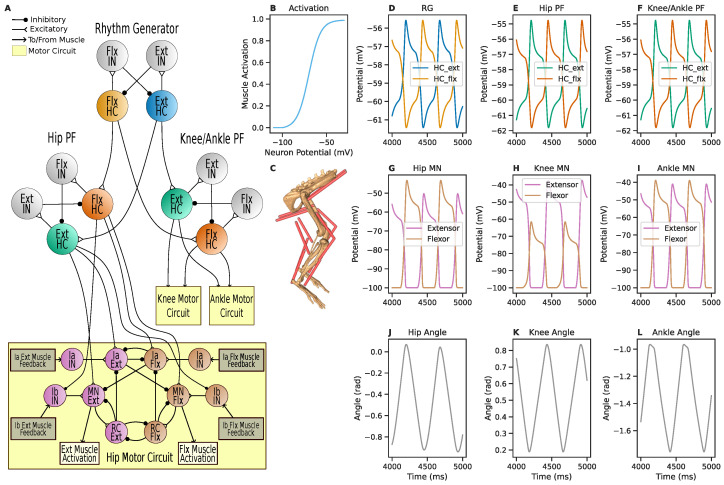
SNS-Toolbox controls a musculoskeletal model of a rat hindlimb. (**A**): Diagram of the neural control network. (**B**): Relationship between motor neuron voltage and muscle activation. (**C**): The musculoskeletal model used in Mujoco [49]. (**D**): Neural activity from the half-center neurons in the central rhythm generator. (**E**,**F**): Neural activity from the hip and knee/ankle pattern formation circuits. (**G**–**I**): Motor neuron activity in the motor circuits for the hip, knee, and ankle. (**J**–**L**): Joint angles of the hip, knee, and ankle. All recordings are shown for a period of 1000 ms, after the model has finished initialization. Pictured are recordings from the elements within the left leg. Right leg recordings are similar, and shown in Figure A1.

An overall network diagram can be found in Figure 6A. The general network structure consists of a two-layer CPG with separate rhythm generation (RG) and pattern formation (PF) layers [69], with each layer comprising of half-center (HC) oscillators [70]. The RG network has two HC neurons which contain voltage-gated ion channels (Equation (14)), which mutually inhibit one another via two non-spiking interneurons (Equation (1)). This network generates the overall rhythmic activity of the legs, and the global speed can be controlled via the level of mutual synaptic inhibition [55].

The HC neurons of the RG network excite the corresponding HC neurons in the PF networks, which are constructed in a similar manner to the RG network. Each PF network shapes the phase from the RG network into the appropriate joint position for a specific joint, with the knee and ankle joints sharing a PF network. The PF networks are also presynaptic to a motor control network for each joint [59,71], where motoneurons (MN) drive the flexor and extensor muscles for each joint and are adjusted by Ia and Ib feedback from the muscles. Neural and synaptic parameter values for the network can be found in Table A4, Table A5 and Table A6.

#### 3.4.2. Biomechanical Model

In Deng et al. [68], the rat hindlimbs were modeled in AnimatLab [37] using simplified box geometry and a pair of linear Hill muscles for each joint. We replicate this model in Mujoco [49] using a three-dimensional model of bone geometry [72] and non-linear Hill muscles (shown in Figure 6C). Mujoco was chosen due to its open-source availability, and its robust internal support for complex muscle-based actuation with a lower computational overhead than OpenSim [73].

All muscles in the model share the same sigmoidal activation curve which converts motoneuron activity to a muscle activation between 0 and 1. This is calculated in the same manner as [74], with the activation sigmoid defined as

(37) $$act=\frac{1}{1+e^{s(x_{offset}-stim)}}+y_{offset},$$

where *s* is the steepness of the sigmoid and stim is the motoneuron potential. All muscle activation parameter values can be found in Table A7, with the resulting curve shown in Figure 6B.

#### 3.4.3. Simulation Results

**Figure A2:**
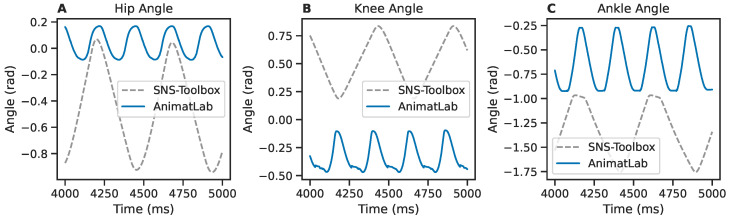
Comparison between simulated motion of the left hindlimb in AnimatLab [37] (solid blue) and Mujoco [49] (dashed grey). (**A**): Hip joint angle. (**B**): Knee joint angle. (**C**): Ankle joint angle. All recordings are shown for a period of 1000 ms, after the model has finished initialization.

The network and mechanical model are simulated for 5000 ms, with data shown in Figure 6. On each step, muscle tensions from Mujoco are first formatted as Ia and Ib feedback for the SNS, then the outputs of the SNS are mapped via Equation (37) into muscle activations for the Mujoco model. The network parameters are exactly the same in this work as in Deng et al. [68], and result in oscillatory motor behavior (the original joint angles from AnimatLab can be seen in Figure A2). The overall leg oscillation occurs at half the frequency of the original model, and the joints exhibit scaled and shifted trajectories. The hip joint oscillates in the range of {−0.94,0.07} radians instead of {−0.09,0.17} radians in the original model, with the knee in {0.19,0.84} radians instead of {−0.47,−0.09} radians and the ankle in {−1.76,−0.97} radians instead of {−0.92,−0.26} radians. Further investigation is needed to determine the sources of these discrepancies in behavior, however a difference is to be expected given the difference in muscle modeling.

## 4. Discussion

In this work, we present SNS-Toolbox, an open-source Python package for simulating synthetic nervous systems. We focus on simulating a specific subset of neural and synaptic models, which allows for improved performance over many existing neural simulators. To the best of our knowledge, the SNS-Toolbox is the only neural simulator available which meets all of the desired functionality for designing synthetic nervous systems. The SNS-Toolbox is not tied to a dedicated graphical user interface, allowing networks to be designed and simulated on embedded systems. Heterogeneous networks of both spiking and non-spiking neurons, as well as chemical and electrical synapses, can also be simulated in real-time on both CPU and GPU hardware. All of these capabilities are also fully available across all major operating systems, including Windows (trademark Microsoft Corporation), MacOS (trademark Apple Corporation), and Linux-based systems.

We find that SNS-Toolbox can simulate networks with hundreds to thousands of neurons in real-time on desktop hardware, and low hundreds of neurons on embedded hardware. The performance is also competitive with other popular neural simulators. Through a simple programming interface, it is relatively simple to combine networks made in SNS-Toolbox with other software. Using ROS [50], we implemented a Braitenberg-inspired [64] neural steering algorithm and controlled navigation of a simulated mobile robot through an environment in Gazebo [36]. We also take an existing SNS network which controlled a musculoskeletal model [68] implemented in AnimatLab [37], and achieved cyclical limb motion after reimplementing the network in SNS-Toolbox and interfacing with Mujoco [49].

One decision we made early in the design process was to provide a simplified design and compilation interface, as well as to build SNS-Toolbox on top of widely used Python numerical processing libraries in order to facilitate use across all computing platforms. This has allowed multiple researchers with varying degrees of programming experience within our laboratories to begin using SNS-Toolbox successfully, as well as an instructional tool in pilot classes on neurorobotics. While other tools, such as ANNarchy [16], achieve higher performance by direct code generation in C++, they do so at the expense of easy cross-platform support. Future work may explore adding additional build systems for different operating systems in order to achieve comparable performance.

In order to allow network simulation on GPUs, multiple backends in SNS-Toolbox are built on top of PyTorch [58]. However, PyTorch has a large infrastructure of features which are currently not supported by the structure of the SNS-Toolbox backend, such as layer-based organization of networks and gradient-based optimization using automatic differentiation. Additionally, models built using the formal PyTorch style are able to be compiled into the C++-adjacent Torchscript, allowing improved simulation performance. Work is currently underway to restructure the PyTorch backend within SNS-Toolbox to allow these benefits.

Previous SNS models have often been made using the software AnimatLab [4,6,7,37], which uses a different workflow than SNS-Toolbox. Within AnimatLab, users have an integrated GUI that contains a rigid-body modeler, canvas for dragging and dropping neurons and synapses into a network, and a plotting window for viewing simulation results. The SNS-Toolbox is designed to focus on the design and simulation of the neural and synaptic dynamics, with the physics simulation and plotting being relegated to external libraries. While this may be less convenient for a user who is either a beginner or is migrating from AnimatLab, we feel that this separation is beneficial as it allows networks made using the SNS-Toolbox to be more extensible to interfacing with other systems. When transitioning from AnimatLab, the primary difference in workflow is that networks in the SNS-Toolbox are described via code instead of drawn. If the transitioning user is familiar with writing code in Python or another similar language, this change is easily managed. The other difficulty when converting from AnimatLab to SNS-Toolbox is when using MuJoCo [49] for physics simulation. As the native muscle model within MuJoCo is different than AnimatLab, we show in Section 3.4 and Figure A2 that the same network with the same parameter values will exhibit different behavior if not tuned to use the new muscle model.

Throughout the design process of SNS-Toolbox, we chose to focus on implementing specific sets of neural and synaptic dynamics. This brings enhanced performance, however it does mean that there is no method for a user to add a new neural or synaptic model to a network which has not been previously defined, without editing the source code for the toolbox itself. One workaround to this issue is to create more complicated models by treating individual non-spiking or spiking neurons as compartments which are connected together as a multi-compartment model, however, in general, we find that this is a limitation for the SNS-Toolbox at this time.

The SNS-Toolbox is currently available as open-source software on GitHub (trademark GitHub incorporated), and has an extensive suite of documentation freely available online. In addition to installing from source, the SNS-Toolbox is also available to install from the Python Package Index (PyPi). All of the features within the toolbox are built on top of standard, widely used Python libraries. As long as these libraries maintain backwards compatibility as they update, or the current versions remain available, the functionality of SNS-Toolbox should remain into the future.

Examples were shown using the SNS-Toolbox to interface with external software systems, particularly ROS [50] and Mujoco [49]. While the primary goal of SNS-Toolbox is a simplified interface which focuses on neural dynamics, other users may find our interface mappings between SNS-Toolbox and other software useful. As such, we intend to release supplementary Python packages which contain helper logic to interface the SNS-Toolbox with other software as we develop them.

Many robots have been built which use SNS networks for control, although these are usually tethered to an off-board computer [6,7] or require non-traditional computer hardware [8] to operate. With the release of SNS-Toolbox, we have two forward-looking hopes. Firstly, that more researchers design and implement synthetic nervous systems for robotic control, and that members of the robotics community will find value in neural simulators which are capable of simulating heterogeneous networks of dynamic neurons.

## Figures and Tables

**Table 1:** Neural Simulator ${}^{*}$ Feature Comparison.

Software	Animat-Lab	NRP	Nengo	SnnTorch	Spyke-Torch	Binds-NET	Brian2	NEU-RON	NEST	ANN-Archy	SNS-Toolbox
GUI Required	X	X									
Real-Time Capable	X		X	X	X	X				X	X
Synaptic Reversal Potentials	X	X ${}^{†}$	X ${}^{†}$				X	X		X	X
Non-Spiking and Spiking	X	X	X				X	X		X	X
Electrical Synapses	X	X ${}^{†}$	X ${}^{†}$				X	X	X	X	X
GPU Support		X ${}^{†}$	X ${}^{†}$	X	X	X	X		X ${}^{‡}$	X ${}^{‡}$	X
Cross Platform ${}^{§}$		X	X	X	X	X	X	X	X		X

^*^ Due to the many simulators available, not all are presented in this table. ^†^ To implement some features, custom code must be implemented which is incompatible with the rest of the Nengo ecosystem. ^‡^ Limited GPU support is currently available. ^*§*^ To be considered cross-platform compatible, the software must be easily run on Linux, MacOS, and Windows.

**Table 2:** Software and hardware specifications.

Item	CPU	GPU	RAM	Python	NumPy	PyTorch	CUDA
Version	AMD Ryzen 9 3900X	NVIDIA RTX2060	32 GB DDR4 2400 MHz	3.8.10	1.21.1	1.9.0	11.5

**Table 3:** Maximum network size.

Backend	Iterative	NumPy	Torch (CPU)	Torch (GPU)	Sparse (CPU)	Sparse (GPU)
Max Dense	11,010	20,010	22,000	7865	151 ${}^{*}$	2510 ${}^{*}$
Max Sparse	158,010	23,010	24,010	7639	17,510 ${}^{*}$	11,120 ${}^{*}$

^*^ Larger networks can be simulated, but compilation takes excessive time.

**Figure A1:**
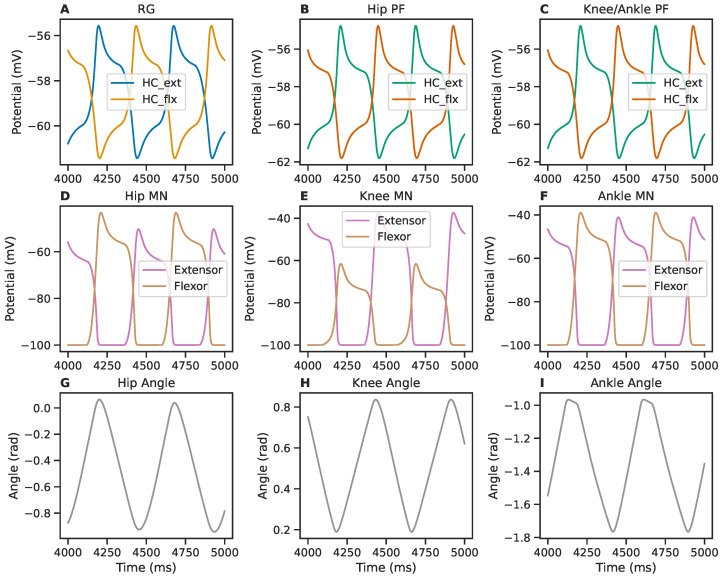
Recordings of elements within the right hindlimb. (**A**): Neural activity from the half-center neurons in the central rhythm generator. (**B**,**C**): Neural activity from the hip and knee/ankle pattern formation circuits. (**D**–**F**): Motor neuron activity in the motor circuits for the hip, knee, and ankle. (**G**–**I**): Joint angles of the hip, knee, and ankle. All recordings are shown for a period of 1000 ms, after the model has finished initialization.

## Data Availability

The software and data presented in this study are openly available https://github.com/wnourse05/SNS-Toolbox (accessed on 9 June 2023), with documentation and detailed instructions provided at https://sns-toolbox.readthedocs.io/en/latest/index.html (accessed on 9 June 2023).

## Abbreviations

The following abbreviations are used in this manuscript:

SNS	Synthetic Nervous System
ANN	Artificial Neural Network
FSA	Functional Subnetwork Approach
ROS	Robotic Operating System
CPU	Central Processing Unit
GPU	Graphics Processing Unit
RG	Rhythm Generation
PF	Pattern Formation
HC	Half-Center
MN	Motoneuron

## Appendix A

**Table A1:** Mobile robot neural parameter values.

Parameter	$V_{\mathrm{rest}}$	$C_{m}$	$G_{m}$
Value	0 mV	5 nF	1 μS

**Table A2:** Mobile robot synaptic parameter values.

Parameter	$E_{\mathrm{in}}$	$E_{\mathrm{ex}}$	$E_{\mathrm{lo}}$	$E_{\mathrm{hi}}$	$G_{\max,\mathrm{angle}}$	$G_{\max,\mathrm{speed}}$
Value	−2.0	5 mV	0 mV	1 mV	0.005 μS	0.0028 μS

**Table A3:** Mobile robot mapping and simulation parameter values.

Parameter	Δt	$D_{\min}$	$D_{\max}$	$K_{\mathrm{ang}}$	$\nu_{\max,\mathrm{lin}}$
Value	1 ms	0.1 m	30 m	5/π	1 m/s

**Table A4:** Rat hindlimb neural parameter values.

Parameter	$C_{m}$	$G_{m}$	$V_{\mathrm{rest}}$	$V_{\mathrm{rest},\mathrm{MN}}$
Value	5 nF	1 μS	−60 mV	−100 mV

**Table A5:** Rat hindlimb sodium channel parameter values.

Parameter	$E_{\mathrm{Na}}$	$S_{m}$	$S_{h}$	$K_{m}$	$K_{h}$	$E_{m}$	$E_{h}$	$\tau_{h.\max}$	$G_{\mathrm{Na}}$
Value	50 mV	0.2	−0.6	1	0.5	−40 mV	−60 mV	350 ms	1.5 μS

**Table A6:** Rat hindlimb synaptic parameter values.

Synapse	$G_{\max}$	$E_{\mathrm{syn}}$	$E_{\mathrm{lo}}$	$E_{\mathrm{hi}}$
HC→IN	2.749 μS	−40 mV	−60 mV	−25 mV
IN→HC	2.749 μS	−70 mV	−60 mV	−25 mV
$RG_{HC}\to PF_{HC}$	0.100 μS	−40 mV	−60 mV	−40 mV
$PF\to MN_{hip,ext}$	2.565 μS	−10 mV	−60 mV	−50 mV
$PF\to MN_{hip,flx}$	3.632 μS	−10 mV	−60 mV	−50 mV
$PF\to MN_{knee,ext}$	4.930 μS	−10 mV	−60 mV	−50 mV
$PF\to MN_{knee,flx}$	1.516 μS	−10 mV	−60 mV	−50 mV
$PF\to MN_{ankle,ext}$	4.054 μS	−10 mV	−60 mV	−50 mV
$PF\to MN_{hip,ext}$	4.522 μS	−10 mV	−60 mV	−50 mV
PF→Ia	0.500 μS	−40 mV	−60 mV	−40 mV
Ia→Ia	0.500 μS	−70 mV	−60 mV	−40 mV
Ia→MN	2.000 μS	−100 mV	−60 mV	−40 mV
MN→RC	0.500 μS	−40 mV	−10 mV	−100 mV
RC→RC	0.500 μS	−70 mV	−60 mV	−40 mV
RC→MN	0.500 μS	−100 mV	−60 mV	−40 mV
RC→Ia	0.500 μS	−70 mV	−60 mV	−40 mV
$Ia_{IN}\to Ia$	0.500 μS	−40 mV	−60 mV	−40 mV
$Ib_{IN}\to MN$	0.590 μS	−10 mV	−60 mV	−40 mV
PF→Ib	2.000 μS	−60 mV	−60 mV	−59 mV

**Table A7:** Rat hindlimb muscle activation and simulation parameter values.

Parameter	$y_{\mathrm{offset}}$	*Stim*	$x_{\mathrm{offset}}$	*s*	Δt
Value	−0.01	[−100,−40] mV	−70 mV	0.1532	0.1 ms
